# Improved prediction value of the CURB-65 score combined with the platelet-to-lymphocyte ratio for mortality in emergency department patients with severe community-acquired pneumonia

**DOI:** 10.3389/fmed.2026.1836427

**Published:** 2026-06-02

**Authors:** Chengtian Lv, Shizhuan Liu, Yongxin Yan, Yaoqu Ye, Yuanmei Gao, Ming Zhou

**Affiliations:** 1 Emergency Department, Yangchun People's Hospital, Yangjiang, China; 2 Department of Critical Care Medicine, Yangchun People's Hospital, Yangjiang, China; 3 Department of Pulmonary and Critical Care Medicine, Yangchun People's Hospital, Yangjiang, China; 4 Emergency Department of Yangjiang People's Hospital, Yangjiang, China; 5 Department of Pulmonary and Critical Care Medicine, The Third Affiliated Hospital of Guangzhou Medical University, Guangzhou, China

**Keywords:** all-cause mortality, CURB-65 score, platelet-to-lymphocyte ratio, prediction value, severe community-acquired pneumonia

## Abstract

**Objective:**

This study aimed to evaluate the value of combining the CURB-65 score with the platelet-to-lymphocyte ratio (PLR) for predicting 30-day all-cause mortality in emergency department patients with severe community-acquired pneumonia (SCAP).

**Methods:**

This retrospective study included 214 adults with SCAP. We compared variables between survivors and nonsurvivors, performed multivariate logistic analysis to identify independent predictors, and used Kaplan–Meier analysis for survival stratification. Receiver operating characteristic (ROC) curve analysis was used to evaluate the predictive performance of CURB-65 alone and in combination with the PLR, the area under the curve (AUC) values of the two correlated ROC curves were compared with those of the DeLong test.

**Results:**

The 30-day mortality was 25.7%. The CURB-65 score was an independent predictor of mortality after multivariable adjustment (OR 2.80, 95% CI 1.87–4.18). Patients were divided into three groups on the basis of their CURB-65 score: low (0–2), medium (3), and high (4–5). The survival rate of the three groups gradually decreased, and the difference in survival curves was statistically significant (*p* < 0.05). Compared with that of CURB-65 alone (AUC = 0.702; 95% CI: 0.629–0.775), the prediction of CURB-65 + PLR was superior (AUC = 0.772; 95% CI: 0.703–0.841), with improved sensitivity (61.0%) and high specificity (81.8%).

**Conclusion:**

Combining the CURB-65 score with the PLR improves the prediction of 30-day mortality in emergency SCAP patients, offering a reference for early risk stratification, but it is not yet a reliable predictor for individual patients.

## 1 Introduction

Community-acquired pneumonia (CAP) is a common infectious disease with persistently high global incidence and mortality rates; it is a leading cause of emergency department visits and hospitalizations and poses a particularly serious threat to older patients and those with underlying comorbidities (24). Despite continuous advancements in antimicrobial therapy and supportive care, patients with severe community-acquired pneumonia (SCAP) often experience rapid clinical deterioration, which is frequently complicated by sepsis, acute respiratory distress syndrome (ARDS), and multiple organ dysfunction syndrome (MODS). Consequently, mortality rates remain substantial, imposing a significant burden on public health systems (1, 2).

In the dynamic and time-sensitive environment of the emergency department, the rapid and accurate identification of high-risk patients and early stratification of their prognoses are critical for optimizing resource allocation, guiding treatment decisions, and ultimately improving patient outcomes. Several pneumonia severity scores are widely employed in clinical practice. Among them, the Confusion, Urea, Respiratory rate, Blood pressure, and Age ≥65 years (CURB-65) score is highly regarded for its simplicity and objective criteria and is consequently recommended by both national and international guidelines for the initial assessment of patients with CAP (3). However, the CURB-65 score, which is based primarily on patients’ physiological parameters, fails to adequately incorporate key information reflecting the underlying systemic inflammatory response and immune status (4). This limitation can compromise its predictive accuracy, particularly in distinguishing patients at intermediate risk, potentially leading to an under- or overestimation of mortality risk.

In recent years, a growing body of research on the pathophysiological mechanisms of CAP has increasingly highlighted the critical role of host inflammatory and immune responses in disease initiation, progression, and ultimately outcomes. Platelets are pivotal coordinators of inflammation as well as innate and adaptive immunity, with both platelets and lymphocytes playing critical roles in the mechanisms underlying inflammatory and infectious diseases (5). The platelet-to-lymphocyte ratio (PLR), an emerging and readily accessible systemic inflammatory biomarker, has demonstrated significant prognostic value in a variety of infectious diseases and cancers (6). Its biological rationale lies in the fact that an elevated platelet count may be associated with infection-triggered inflammatory cascades and a prothrombotic state, whereas lymphocytopenia reflects a state of stress-induced immunosuppression. By integrating these two dimensions, the PLR provides a more comprehensive assessment of the balance between proinflammatory and immune statuses (7). The PLR has been validated as a valuable tool for the diagnosis and prognosis prediction of conditions such as sepsis, acute pulmonary embolism, AECOPD, and COVID-19 (8–11). However, whether the combination of this novel inflammatory marker with the established CURB-65 score can more accurately predict short-term mortality risk, specifically 30-day mortality after admission, in SCAP patients remains to be further studied.

Therefore, the purpose of this study was to explore the ability of the combination of the CURB-65 score and PLR to predict 30-day all-cause mortality in patients with SCAP in the emergency department and to provide a clinical basis for emergency doctors to identify high-risk patients with SCAP early and implement more appropriate intervention measures.

## 2 Methods

A total of 214 emergency department patients with SCAP who were admitted to Yangchun People’s Hospital in Guangdong Province between July 2024 and October 2025 were enrolled in this study. The diagnosis of SCAP was made according to the criteria defined by the American Thoracic Society (12). The inclusion criteria included age ≥18 years, clinical presentation consistent with CAP (e.g., cough and upper respiratory symptoms) with either community acquisition or onset within 48 h of admission, and a definitive diagnosis of SCAP.

The diagnostic criteria for SCAP included meeting at least one major criterion—either the need for mechanical ventilation via endotracheal intubation or a vasopressor requirement for septic shock refractory to fluid resuscitation—or fulfilling three or more of the following minor criteria: a respiratory rate ≥ 30 breaths/min, a PaO₂/FiO₂ ratio ≤ 250 mmHg, a BUN concentration ≥ 20 mg/dL, leukopenia (WBC count < 4,000/μL) from no other cause, thrombocytopenia (platelet count < 100 × 10^9^/L), hypothermia (core temperature < 36 °C), new-onset confusion/disorientation, or new radiographic lung infiltrates consistent with CAP (12).

Patients who were under 18 years of age, had incomplete medical records, had comorbid hematological malignancies, presented with leukopenia and/or neutropenia, or had any immunosuppression-causing condition (including chronic immunosuppressive therapy, solid organ transplant status, postplenectomy status, or AIDS) were excluded from the study. For data analysis, the cohort was dichotomized by 30-day survival status into survivors and nonsurvivors, and regression analysis was used to identify associated risk factors. All patients were managed following the standard therapeutic guidelines of the ATS/IDSA.

Patient sex, age, respiration, blood pressure, basic disease information, total platelet count, total lymphocyte count, hemoglobin level, serum C-reactive protein level, procalcitonin level, urea nitrogen level, creatinine level, serum lactic acid level, and CURB-65 score in the early stage of admission (within 24 h) were recorded, and the ratio of platelets to lymphocytes was calculated.

### 2.1 Statistical analysis

Data analysis was performed by using SPSS version 25.0 and Free Statistics software version 2.3. Normally distributed continuous data are presented as the mean ± standard deviation (SD), and comparisons between the two groups were made by using the independent samples t test. Nonnormally distributed continuous data are expressed as medians with interquartile ranges [*M* (P25, P75)], and the Mann–Whitney U test was used for intergroup comparisons. Categorical data are presented as numbers and percentages, and comparisons were conducted by using the Pearson chi-square (χ^2^) test. Multivariate logistic regression analysis was performed to identify independent risk factors for 30-day mortality. The results are presented as odds ratios (ORs) with 95% confidence intervals (CIs). In the multivariable logistic regression analysis, the PLR was subjected to a linear transformation by dividing by 100 (i.e., the variable used in the model was PLR/100), and the serum creatinine level and ALT level were subjected to a linear transformation by dividing by 10 to mitigate the influence of extreme values and enhance the interpretability of the model coefficients. Consequently, the odds ratio (OR) for the PLR in the model represents the change in mortality risk associated with each 100-unit increase in the original PLR value, and the OR for the serum creatinine and ALT in the model represents the change in mortality risk associated with each 10-unit increase in the original serum creatinine and ALT values. Collinearity among the independent variables was assessed by using the variance inflation factor (VIF), and a VIF value of less than 2 was considered to indicate the absence of significant collinearity. Survival analysis was conducted using Kaplan–Meier methods with log-rank tests. The predictive performance of Model 1 (CURB-65 alone) and Model 2 (CURB-65 combined with PLR) was evaluated using receiver operating characteristic (ROC) curves. The area under the curve (AUC) values of the two correlated ROC curves were compared with those of the DeLong test. A two-sided *p* value of less than 0.05 was considered to indicate statistical significance.

## 3 Results

### 3.1 Baseline characteristics and clinical indicators

**Figure 1:**
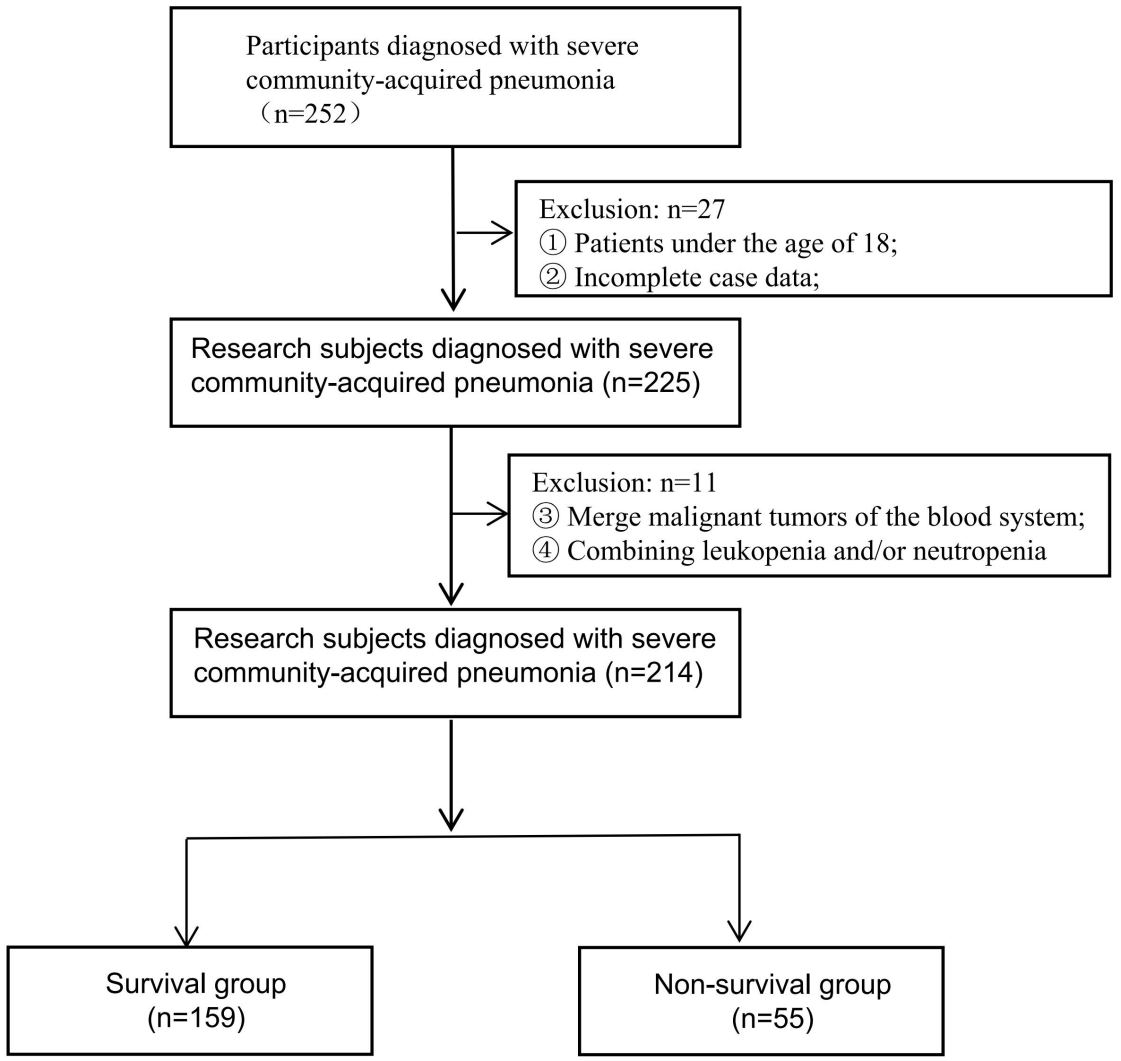
The flowchart of this study.

A total of 214 emergency department patients with SCAP were included in this study, of whom 55 patients (25.7%) died within 30 days of admission. A flow chart of the inclusion and exclusion criteria is presented in Figure 1. The baseline clinical data of the participants in the survivor and nonsurvivor groups are shown in Table 1. No significant differences were observed between the two groups in terms of age, sex distribution, heart rate, Glasgow Coma Scale score, or serum sodium level (*p* > 0.05). However, the prevalence of preexisting chronic cardiovascular disease (56.0% vs. 27.3%, *p* < 0.05) and nervous system disease (47.2% vs. 5.5%, *p* < 0.05) was significantly greater in the survivor group.

**Table 1:** Baseline characteristics of the participants.

Variables	Total (*n* = 214)	Survivors (*n* = 159)	Nonsurvivors (*n* = 55)	*p* value
Age, years, Mean ± SD	69.2 ± 13.6	68.9 ± 13.3	69.9 ± 14.5	0.641
Sex, *n* (%)				0.225
Female	64 (29.9)	44 (27.7)	20 (36.4)	
Male	150 (70.1)	115 (72.3)	35 (63.6)	
Chronic cardiovascular disease, *n* (%)	104 (48.6)	89 (56)	15 (27.3)	< 0.001*
Nervous system disease, *n* (%)	78 (36.4)	75 (47.2)	3 (5.5)	< 0.001*
Heart rate, beats/min, Mean ± SD	112.6 ± 48.6	111.7 ± 53.9	115.2 ± 28.2	0.648
Respiratory rate, breaths/min, Mean ± SD	27.8 ± 6.6	27.2 ± 6.7	29.5 ± 6.0	0.027*
Systolic blood pressure, mmHg, Mean ± SD	129.3 ± 32.2	132.9 ± 31.2	119.1 ± 33.1	0.006*
Diastolic blood pressure, mmHg, Mean ± SD	76.8 ± 33.6	79.4 ± 36.9	69.0 ± 19.8	0.047
Glasgow score, Median (IQR)	11.0 (7.0, 13.0)	11.0 (7.0, 13.0)	11.0 (6.0, 13.0)	0.374
Na^+^, mmol/L, Mean ± SD	139.7 ± 8.7	139.7 ± 7.2	140.0 ± 12.0	0.81
K^+^, mmol/L, Median (IQR)	3.6 (3.1, 4.2)	3.6 (3.1, 4.0)	4.0 (3.3, 4.7)	0.01*
Lactic acid, mmol/L, Median (IQR)	2.3 (1.4, 4.4)	2.0 (1.3, 4.1)	3.2 (2.0, 5.4)	0.003*
WBCs, 10^9^/L, Mean ± SD	14.1 ± 7.4	14.0 ± 7.4	14.3 ± 7.4	0.825
RBCs, 10^12^/L, Mean ± SD	3.9 ± 1.0	4.1 ± 0.9	3.6 ± 1.2	0.007*
Hb, g/L, Mean ± SD	110.5 ± 27.6	113.7 ± 24.4	101.4 ± 33.8	0.004*
Platelets, 10^9^/L, Mean ± SD	217.0 ± 106.2	222.3 ± 96.8	201.4 ± 129.4	0.208
Lymphocytes, 10^9^/L, Median (IQR)	0.8 (0.5, 1.2)	0.9 (0.6, 1.4)	0.5 (0.4, 0.8)	< 0.001*
Neutrophils, 10^9^/L, Median (IQR)	12.9 ± 8.5	12.6 ± 9.1	13.7 ± 6.5	0.414
Platelet-to-lymphocyte ratio, Median (IQR)	236.6 (158.5, 367.4)	216.8 (142.8, 321.7)	280.7 (213.3, 500.7)	< 0.001*
ALT, U/L, Median (IQR)	26.0 (17.0, 52.0)	23.0 (16.0, 44.5)	46.0 (22.5, 94.0)	0.002*
AST, U/L, Median (IQR)	39.0 (23.2, 80.2)	34.0 (22.5, 58.5)	68.0 (32.0, 166.0)	< 0.001*
ALB, g/L, Mean ± SD	31.8 ± 6.3	32.8 ± 6.3	29.0 ± 5.4	< 0.001*
C-reactive protein, mg/L, Median (IQR)	76.6 (25.7, 187.0)	71.5 (22.0, 186.9)	95.1 (54.2, 181.1)	0.085
PCT, ng/mL, Median (IQR)	1.0 (0.2, 6.2)	0.7 (0.2, 6.5)	2.0 (0.9, 6.0)	0.007*
Blood urea nitrogen, mmol/L, Median (IQR)	8.2 (5.5, 13.7)	7.3 (5.1, 11.4)	11.0 (7.8, 20.0)	< 0.001*
Serum creatinine, mmol/L, Median (IQR)	95.0 (65.0, 174.8)	87.0 (65.0, 132.5)	133.0 (73.5, 262.5)	0.005*
CURB-65 score, *n* (%)				< 0.001*
1	29 (13.6)	29 (18.2)	0 (0)	
2	48 (22.4)	39 (24.5)	9 (16.4)	
3	82 (38.3)	60 (37.7)	22 (40)	
4	43 (20.1)	28 (17.6)	15 (27.3)	
5	12 (5.6)	3 (1.9)	9 (16.4)	

*p < 0.05 indicates statistical significance.

Clinically, the nonsurvivor group presented more severe physiological derangements, including a higher respiratory rate (29.5 ± 6.0 vs. 27.2 ± 6.7 breaths/min, *p* < 0.05), lower systolic blood pressure (119.1 ± 33.1 vs. 132.9 ± 31.2 mmHg, *p* < 0.05), and lower diastolic blood pressure (69.0 ± 19.8 vs. 79.4 ± 36.9 mmHg, *p* < 0.05). They also had significantly higher serum potassium levels (4.0 [IQR 3.3–4.7] vs. 3.6 [IQR 3.1–4.0] mmol/L, *p* < 0.05).

The levels of critical laboratory markers of tissue perfusion, inflammation, and organ dysfunction were markedly worse in nonsurvivors. Their lactate levels were significantly elevated (3.2 [IQR 2.0–5.4] vs. 2.0 [IQR 1.3–4.1] mmol/L, *p* < 0.05). Hematological analysis revealed that the nonsurvivors had significantly lower red blood cell counts (3.6 ± 1.2 vs. 4.1 ± 0.9 × 10^12^/L, *p* < 0.05), hemoglobin levels (101.4 ± 33.8 vs. 113.7 ± 24.4 g/L, *p* < 0.05), and most notably, lymphocyte counts (0.5 [IQR 0.4–0.8] vs. 0.9 [IQR 0.6–1.4] × 10^9^/L, *p* < 0.05). Consequently, the PLR (280.7 [IQR 213.3–500.7] vs. 216.8 [IQR 142.8–321.7], *p* < 0.05) was significantly greater in the nonsurvivors.

Evidence of hepatic and renal impairment was also more pronounced in the nonsurvivor group, with higher levels of ALT (46.0 [IQR 22.5–94.0] vs. 23.0 [IQR 16.0–44.5] U/L, *p* = 0.002), AST (68.0 [IQR 32.0–166.0] vs. 34.0 [IQR 22.5–58.5] U/L, *p* < 0.05), blood urea nitrogen (11.0 [IQR 7.8–20.0] vs. 7.3 [IQR 5.1–11.4] mmol/L, *p* < 0.05), and serum creatinine (133.0 [IQR 73.5–262.5] mmol/L, *p* < 0.05). In contrast, serum ALB levels were significantly lower in nonsurvivors (29.0 ± 5.4 vs. 32.8 ± 6.3 g/L, *p* < 0.05). In addition, procalcitonin levels were significantly elevated in nonsurvivors (2.0 [IQR 0.9–6.0] vs. 0.7 [IQR 0.2–6.5] ng/mL, *p* < 0.05). Finally, the CURB-65 score was strongly associated with mortality. The distribution of the CURB-65 scores significantly differed among the groups (*p* < 0.05).

### 3.2 Multivariable logistic regression analysis of risk factors for survival outcomes

Multivariate logistic regression analysis was performed to identify independent risk factors for survival outcomes in patients with SCAP. The results are summarized in Table 2. After adjusting for other covariates, RBCs, lymphocytes, the PLR, lactic acid levels, serum creatinine levels, blood urea nitrogen levels, serum ALB levels, serum ALT levels, and the CURB-65 score were identified as statistically significant independent predictors. In terms of hematological parameters, a lower lymphocyte count (0.11, 95% CI 0.04–0.29; *p* < 0.05) was closely related to increased mortality, whereas the platelet count and mortality were not significantly different (*p* > 0.05); however, the PLR (1.35, 95% CI 1.16–1.58; *p* < 0.05) was still a significant independent predictor. PLR was subjected to a linear transformation by dividing by 100.

**Table 2:** Multivariate logistic regression analysis of risk factors for survival outcomes in patients with SCAP.

Variable	OR (95% CI)	*p* value
WBCs, 10^9^/L	1.00 (0.96 ~ 1.05)	0.866
RBCs, 10^12^/L	0.67 (0.48 ~ 0.92)	0.014*
Neutrophils, 10^9^/L	1.01 (0.98 ~ 1.05)	0.504
Platelets, 10^9^/L	1.00 (0.99 ~ 1.00)	0.119
Lymphocytes, 10^9^/L	0.11 (0.04 ~ 0.29)	<0.001*
Platelet-to-lymphocyte ratio**	1.35 (1.16 ~ 1.58)	<0.001*
Lactic acid, mmol/L	1.13 (1.04 ~ 1.23)	0.005*
Serum creatinine, mmol/L**	1.02 (1.00 ~ 1.03)	0.045*
Blood urea nitrogen, mmol/L	1.06 (1.03 ~ 1.09)	<0.001*
ALB, g/L	0.90 (0.85 ~ 0.95)	<0.001*
ALT, U/L**	1.05 (1.01 ~ 1.08)	0.005*
C-reactive protein, mg/L	1.00 (1.00 ~ 1.00)	0.314
PCT, ng/mL	1.00 (0.98 ~ 1.01)	0.732
CURB-65 score	2.80 (1.87 ~ 4.18)	<0.001*

*p < 0.05 indicates statistical significance. ^**^The platelet-to-lymphocyte ratio (PLR) underwent a linear transformation divided by 100 (i.e., the variables used in the analysis were PLR/100), and serum creatinine and ALT levels underwent a linear transformation divided by 10.

Key metabolic and organ function markers also demonstrated predictive value. Elevated serum lactic acid levels (OR 1.13, 95% CI 1.04–1.23; *p* < 0.05), blood urea nitrogen (BUN) levels (OR 1.06, 95% CI 1.03–1.09; *p* < 0.05), and serum creatinine levels (OR 1.02, 95% CI 1.00–1.03; *p* < 0.05) were significant risk factors. Conversely, higher serum albumin (ALB) levels were a protective factor (OR 0.90, 95% CI 0.85–0.95; *p* < 0.05). Additionally, the alanine aminotransferase (ALT) level was a significant positive predictor (OR 1.05, 95% CI 1.01–1.08; *p* < 0.05). Serum creatinine and ALT levels were subjected to linear transformation by dividing by 10.

Finally, the prognostic utility of the established clinical severity CURB-65 score was confirmed, with a one-point increase associated with a 2.80-fold increase in mortality risk (OR 2.80, 95% CI 1.87–4.18; *p* < 0.05). The VIF values for all included variables were less than 2 (range: 1.04 to 1.79), indicating that collinearity was not a concern in our model. As shown in the Supplementary Table S1, variables such as white blood cell count, neutrophil count, platelet count, C-reactive protein level, and procalcitonin level did not have independent predictive value in the multivariable model (*p* > 0.05).

### 3.3 Kaplan–Meier curve analysis to evaluate survival differences between the CURB-65 score and PLR stratification

**Figure 2:**
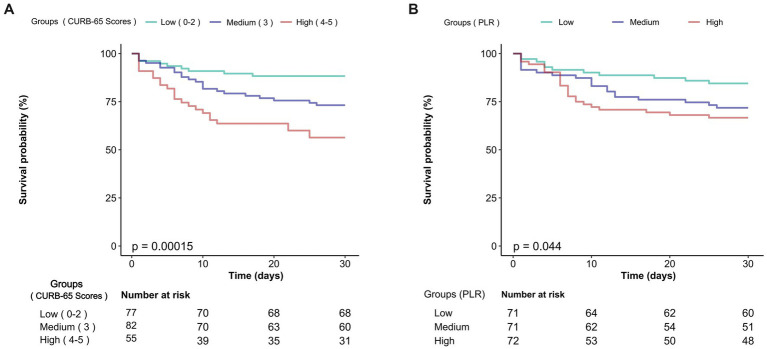
Kaplan–Meier curve analysis to evaluate survival differences between the CURB-65 score and PLR stratification. **(A)** Patients were divided into three groups according to the CURB-65 score: low-level (score 0–2), *n* = 77; intermediate-level (score 3), *n* = 82; and high-level (score 4–5), *n* = 55. The survival probability progressively decreased across these groups. The survival curves significantly differed across the three groups (*p* < 0.05). **(B)** According to the variable PLR, the data were divided into three equal groups, namely, the low-level group (14.19 ~ 185.82) (*n* = 71), the medium-level group (185.83 ~ 305.55) (*n* = 71), and the high-level group (305.56 ~ 3,000) (*n* = 72). The survival curves significantly differed across the three groups (*p* = 0.044).

The prognostic value of the CURB-65 score for 30-day survival was assessed using Kaplan–Meier analysis. The CURB-65 score was divided into three groups, of which 0–2 represented the low score group, 3 represented the medium score group, and 4–5 represented the high score group. As shown in Figure 2A. Patients were categorized into three groups: low-level (score 0–2) (*n* = 77), intermediate-level (score 3) (*n* = 82), and high-level (score 4–5) (*n* = 55). The survival probability progressively decreased across these groups. The survival curves significantly differed across the three groups (log-rank test, *p* < 0.05). With increasing CURB-65 score, the survival rate decreased significantly, from 88.31% in the low-score group to 56.36% in the high-score group. These results indicate that the CURB-65 score can be used as a prognostic tool for the risk stratification of patients with SCAP in the emergency department and that the risk of death is greater in the high-risk group.

As shown in Figure 2B. The impact of PLR stratification on 30-day survival was evaluated using Kaplan–Meier analysis. According to the variable PLR, the data are divided into three equal groups, namely, the low-level group (14.19 ~ 185.82) (*n* = 71), the medium-level group (185.83 ~ 305.55) (*n* = 71), and the high-level group (305.56 ~ 3,000) (*n* = 72). The survival curves significantly differed across the three groups (log-rank test, *p* < 0.05). A stepwise decrease in survival probability was observed with increasing PLR, with the low-PLR group having the highest cumulative survival rate, followed by the medium-PLR group and then the high-PLR group. The number in the risk table indicates that by the end of the study (day 30), 60, 51, and 48 patients remained alive in the low, medium, and high PLR groups, respectively. With increasing PLR, the survival rate decreased significantly, from 84.51% in the low-PLR group to 66.67% in the high-PLR group. These results indicate that the higher the PLR is and the higher the 30-day mortality risk of patients with SCAP.

### 3.4 ROC curve analysis of the predictive value of the CURB-65 score and the CURB-65 score combined with the PLR for 30-day all-cause mortality in SCAP patients

**Figure 3:**
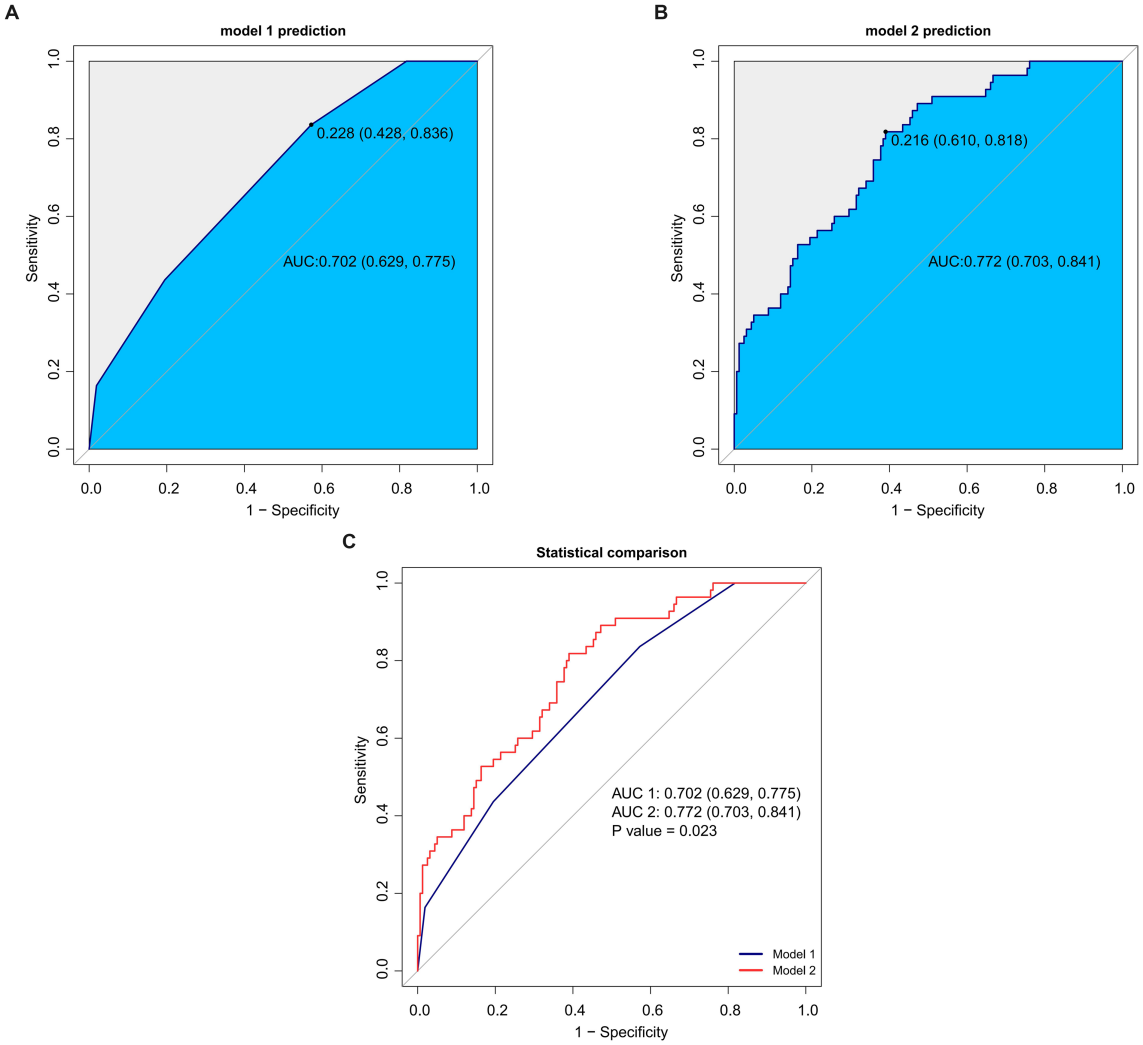
ROC curve analysis evaluating the performance of CURB-65 and CURB-65 combined with the PLR in predicting 30-day all-cause mortality among SCAP patients: **(A)** Model 1 (CURB-65 alone); **(B)** Model 2 (CURB-65 + PLR); **(C)** The DeLong test confirmed a significant difference in AUC between the two models (*p* = 0.023). OR, odds ratio; AUC, area under the curve; CI, confidence interval; PLR, platelet-to-lymphocyte ratio; SCAP, severe community-acquired pneumonia.

ROC analysis was performed to compare the performance of the two models for predicting 30-day mortality. In Model 1, the predictive value of the CURB-65 score alone was significant, with an area under the curve (AUC) of 0.702 (95% CI: 0.629–0.775). At its optimal cutoff value (0.228), the sensitivity was 42.8%, and the specificity was 83.6%, as shown in Figure 3A. Model 2, which integrated the CURB-65 categories with the platelet-to-lymphocyte ratio (PLR), demonstrated a significantly improved predictive accuracy, with an AUC of 0.772 (95% CI: 0.703–0.841). At its optimal cutoff value (0.216), the sensitivity was 61.0%, and the specificity was 81.8%, as shown in Figure 3B. The DeLong test confirmed a statistically significant difference between the two models (*p* < 0.05), as shown in Figure 3C.

## 4 Discussion

This retrospective study included 214 emergency department patients with SCAP. CURB-65 alone had good predictive value for 30-day mortality (AUC = 0.702), and its combination with the PLR significantly improved prognosis (AUC = 0.772; *p* < 0.05).

In this study, the AUC value of the CURB-65 score for predicting 30-day mortality was 0.702, which was lower than the value of 0.83 reported by Aliye Gamze Calis et al. (13). This difference may be due to the difference in the study population. The study of Aliye Gamze Calis et al. included all community-dwelling patients with pneumonia, whereas this study focused specifically on patients with severe community-acquired pneumonia in the emergency department.

Survivor–nonsurvivor comparisons revealed high-risk SCAP features: tachypnea, hypotension, elevated lactate, severe systemic inflammation (lymphopenia, elevated PLR), organ dysfunction, and hypoalbuminemia. These findings are consistent with the pathophysiology of severe infection (14). Consistent with the report of Zaki ha et al. (15) our study revealed that the CURB-65 score is an independent predictor of 30-day mortality in patients with emergency SCAP, validating its role as a core emergency risk stratification tool (15). Accumulating evidence has demonstrated that novel hematological and inflammatory-derived ratios are simple, readily available, and reliable prognostic biomarkers for evaluating disease severity and clinical outcomes in patients with pneumonia. In a 2025 study by Ari et al. involving 120 pediatric intensive care unit (PICU) patients with CAP, C-reactive protein (CRP)/mean platelet volume (MPV) ratio and the lactate/albumin ratio were significantly associated with mortality. Notably, the lactate/albumin ratio yielded better predictive performance for mortality in pediatric patients with CAP than did lactate alone (16). These findings indicate that hematological and inflammatory ratio markers can reflect subtle alterations in systemic inflammation and provide incremental prognostic information for CAP patients, which is insufficiently captured by conventional clinical indicators. Our findings align with a growing body of evidence on the prognostic value of inflammatory ratios in pneumonia and sepsis (17–19). Lymphopenia, a key component of the PLR, is a recognized marker of stress-induced immunosuppression, a pathological state that predisposes patients to secondary infections and mortality (20, 21). Thrombocytosis, or a relative elevation in the platelet count within the ratio, can be part of the acute phase response (22). Thus, the PLR serves as a composite index of both proinflammatory (platelet) and immunosuppressive (lymphocyte) pathways.

Although several studies have explored the predictive value of CURB-65 combined with inflammatory markers for SCAP prognosis, most of them have focused on inpatients in general wards or ICUs, whereas our study specifically targeted emergency department patients with SCAP (15, 23). Patients in the emergency department have the characteristics of acute onset, complex conditions, and an urgent need for triage, which are significantly different from those of inpatients. Our research provides a basis for the application of the CURB-65 + PLR combination model in emergency department SCAP triage and provides emergency department doctors with a simple, fast, and cost-effective prediction tool, which is consistent with the clinical needs of emergency triage.

On the basis of clinical practice in the emergency department, low sensitivity in early risk stratification of severe pneumonia means that a portion of high-risk patients may be missed during the diagnostic process; however, insufficient specificity may lead to over-triage in the emergency department, improper allocation of resources in the intensive care unit, and waste of medical resources. With respect to the CURB-65 score alone, at its optimal cutoff value, the sensitivity was 42.8%, and the specificity was 83.6%. However, when the CURB-65 score was combined with the PLR for predicting 30-day mortality in patients with SCAP, at its optimal cutoff value, the sensitivity was 61.0%, and the specificity was 81.8%. The emergency department SCAP patients included in this study were mostly elderly and had multiple underlying diseases, dull inflammatory responses, and atypical clinical manifestations, which weakened the predictive power of inflammatory indicators such as the PLR to some extent and were important reasons for the overall sensitivity limitation. The CURB-65 score reflects clinical severity and organ dysfunction, whereas the PLR reflects the underlying intensity of the host’s inflammatory response. The integration of these two dimensions—clinical presentation and dysregulated immunity—appears to offer a more holistic assessment of patient risk.

### 4.1 Limitations

First, because this was a single-center study with a limited sample size, the model has not yet undergone internal or external validation, and its robustness, universality, and potential clinical applicability still need further validation. Second, although we corrected for known confounding factors by multivariate logistic analysis, there may still be unmeasured or unknown confounding factors, which may affect the reliability of the results. In addition, the retrospective design itself cannot confirm causal relationships and can reveal only the statistical correlation between variables. In addition, the limited sample size may limit the statistical validity and affect the universality of the conclusions. Multicenter and prospective studies need to verify these findings in the future. Finally, because of its low sensitivity (42.8%), the combined model is better because its sensitivity is higher (61.0%). The combined model is better than CURB-65 alone, but it is still far from a reliable predictor for individual patients.

## 5 Conclusion

In conclusion, the CURB-65 score was an independent predictor of 30-day all-cause mortality in patients with emergency SCAP. Compared with the CURB-65 score alone, the CURB-65 score combined with the PLR is a better predictor of 30-day mortality in patients with SCAP in the emergency department and provides a reference for improving early risk stratification and guiding clinical decision-making, but it cannot yet become a reliable predictor for individual patients.

## Data Availability

The raw data supporting the conclusions of this article will be made available by the authors, without undue reservation.
