# Supplementary material for: Improved prediction value of the CURB-65 score combined with the platelet-to-lymphocyte ratio for mortality in emergency department patients with severe community-acquired pneumonia
Source: Front Med (Lausanne). 2026 Jun 2;13:1836427. doi: 10.3389/fmed.2026.1836427 (PMC13268932; doi:10.3389/fmed.2026.1836427)
Supplement: Supplementary file 1 [file Table_1.doc]

**Attached materials:** Collinearity analysis of the platelet-to-lymphocyte ratio and other covariates.

| Term1 | Coeff1 | Change.percentage1 | Term2 | Coeff2 | Change.percentage2 | GVIF | DF | GVIF^(1/(2*Df))^ | collinearity | Select | Select.VIF |
| --- | --- | --- | --- | --- | --- | --- | --- | --- | --- | --- | --- |
| Crude | 0 | Ref. | Full | 0 | Ref. | 1.329 | 1 | 1.153 | 0 | Ref. | Ref. |
| RBCs | 0 | 2.6 | RBCs | 0 | 0.2 | 1.12 | 1 | 1.058 | 0 | No | No |
| Lymphocytes | 0 | -57.6 | Lymphocytes | 0 | 75.8 | 1.333 | 1 | 1.155 | 0 | Yes | Yes |
| Lactic acid | 0 | 7.5 | Lactic acid | 0 | -2.8 | 1.282 | 1 | 1.132 | 0 | No | No |
| Serum creatinine | 0 | 3.1 | Serum creatinine | 0 | 2.3 | 3.343 | 1 | 1.828 | 0 | No | No |
| Blood urea nitrogen | 0 | 0.3 | Blood urea nitrogen | 0 | 2.1 | 3.484 | 1 | 1.866 | 0 | No | No |
| ALB | 0 | -0.8 | ALB | 0 | -0.8 | 1.124 | 1 | 1.06 | 0 | No | No |
| ALT | 0 | 4.8 | ALT | 0 | -3.3 | 1.17 | 1 | 1.082 | 0 | No | No |
| CURB-65 score | 0 | -6.9 | CURB-65 score | 0 | 5 | 1.295 | 4 | 1.033 | 0 | No | No |
