# Supplementary material for: Improved prediction value of the CURB-65 score combined with the platelet-to-lymphocyte ratio for mortality in emergency department patients with severe community-acquired pneumonia
Source: Front Med (Lausanne). 2026 Jun 2;13:1836427. doi: 10.3389/fmed.2026.1836427 (PMC13268932; doi:10.3389/fmed.2026.1836427)
Supplement: Supplementary file 2 [file Table_2.doc]

Supplementary Table S2. Net reclassification improvement (NRI) and integrated discrimination improvement (IDI) results for Model 1 vs. Model 2

| Old.M | New.M.[0,0.2) | New.M.[0.2,0.4) | New.M.[0.4,1] | reclassified.% |
| --- | --- | --- | --- | --- |
|  | Combined Data | Combined Data | Combined Data | Combined Data |
| [0,0.2) | 68 | 5 | 4 | 12 |
| [0.2,0.4) | 28 | 76 | 21 | 39 |
| [0.4,1] | 0 | 0 | 12 | 0 |
|  | Outcome: absent | Outcome: absent | Outcome: absent | Outcome: absent |
| [0,0.2)1 | 62 | 5 | 1 | 9 |
| [0.2,0.4)1 | 25 | 50 | 13 | 43 |
| [0.4,1]1 | 0 | 0 | 3 | 0 |
|  | Outcome: present | Outcome: present | Outcome: present | Outcome: present |
| [0,0.2)2 | 6 | 0 | 3 | 33 |
| [0.2,0.4)2 | 3 | 26 | 8 | 30 |
| [0.4,1]2 | 0 | 0 | 9 | 0 |

| Item | Estimate | Std.Error | Lower | Upper | type |
| --- | --- | --- | --- | --- | --- |
| NRI | 0.183 | 0.141 | -0.085 | 0.467 | category |
| NRI+ | 0.145 | 0.158 | -0.302 | 0.318 | category |
| NRI- | 0.038 | 0.149 | -0.116 | 0.453 | category |
| Pr(Up|Case) | 0.2 | 0.089 | 0.032 | 0.375 | category |
| Pr(Down|Case) | 0.055 | 0.113 | 0 | 0.408 | category |
| Pr(Down|Ctrl) | 0.157 | 0.135 | 0 | 0.504 | category |
| Pr(Up|Ctrl) | 0.119 | 0.035 | 0.013 | 0.153 | category |
| NRI1 | 0.33 | 0.12 | 0.063 | 0.568 | diff |
| NRI+1 | 0.091 | 0.07 | -0.076 | 0.192 | diff |
| NRI-1 | 0.239 | 0.098 | 0.025 | 0.42 | diff |
| Pr(Up|Case)1 | 0.382 | 0.081 | 0.184 | 0.49 | diff |
| Pr(Down|Case)1 | 0.291 | 0.089 | 0.096 | 0.455 | diff |
| Pr(Down|Ctrl)1 | 0.365 | 0.103 | 0.138 | 0.535 | diff |
| Pr(Up|Ctrl)1 | 0.126 | 0.023 | 0.075 | 0.16 | diff |

| **list.NDI_IDI** |
| --- |
| NRI(Categorical) [95% CI]:0.1832[0.0317-0.3347]; p-value:0.01779 |
| NRI(Continuous) [95% CI]:0.3318[0.0356-0.6281]; p-value:0.02814 |
| IDI [95% CI]:0.0766[0.026-0.1271]; p-value:0.00299 |
